# Phosphodiesterase-5 Expression in Buccal Mucosa of Patients with Erectile Dysfunction One Year after Radical Prostatectomy

**DOI:** 10.3390/jpm14080869

**Published:** 2024-08-17

**Authors:** Juan García-Cardoso, José J. Zamorano-León, Carmen González-Enguita, Carlos Simón, Rodrigo Jiménez-García, Ana López-de-Andrés, Natividad Cuadrado-Corrales, David Carbantes-Alarcon, Carlos Hugo Martínez-Martínez, Khaoula Zekri-Nechar

**Affiliations:** 1 Urology Deparment, Hospital Universitario Fundación Jiménez Díaz, 28040 Madrid, Spain; 2 Public Health and Maternal-Child Health, School of Medicine, Universidad Complutense de Madrid, 28040 Madrid, Spain; 3 Medicine Department, School of Medicine, Universidad Complutense de Madrid, 28040 Madrid, Spain; 4 Health Research Institute of the Hospital Clínico San Carlos (IdISSC), 28040 Madrid, Spain

**Keywords:** buccal mucosa, erectile dysfunction, interleukins, phosphodiesterase 5, nitric oxide system

## Abstract

(1) Background: Radical prostatectomy has a high incidence of erectile dysfunction (ED). The aim was to determine if the expression of the nitric oxide synthase-3/soluble guanylate cyclase/phosphodiesterase 5 axis could be detected in buccal mucosa and if it could be differently expressed in patients with and without ED; (2) Methods: Erectile function from 38 subjects subjected to prostatectomy was evaluated using the International Index of Erectile Function-Erectile Function Domain before and one year after surgery. Nitric oxide synthase (NOS3), β1-subunit of soluble guanylate cyclase (sGC), phosphodiesterase-5 (PDE-5) expressions, and interleukin-6 and interleukin-10 content were measured in the buccal mucosa. PDE5A rs3806808 gene polymorphism was genotyped; (3) Results: One year after prostatectomy, 15 patients had recovered functional erection, and 23 showed ED. NOS3, β1-sGC, interleukin-6, and interleukin-10 expressions were not different between patients with and without ED after radical prostatectomy. Buccal mucosa levels of PDE-5 were higher in patients with ED compared to those who recovered erectile functionality. There were no differences found in the genotype of PDE5A polymorphism; (4) Conclusions: One year after prostatectomy, patients with ED had higher PDE5 levels in their buccal mucosa than patients who had recovered erectile function. Rs3806808 PDE5A gene polymorphism was not associated with increased PDE5 expression in buccal mucosa.

## 1. Introduction

Radical prostatectomy is a common therapeutic approach to treating localized prostatic cancer [1]. Although its oncological results are very good, erectile dysfunction (ED) is a well-identified post-surgical complication [2]. It has been reported that the incidence of ED long-term after prostatectomy ranges between 14–90% [3,4].

ED-related prostatectomy has been related to cavernous nerve damage and cytokines production [5]. It has been widely studied that factors such as age, toxic habits like alcohol consumption or smoking, and diseases such as diabetes and cardiovascular and neurological pathologies play a crucial role in the appearance and progression of erectile dysfunction [6,7]. Additionally, these factors may modulate the response to pharmacological treatment of erectile dysfunction and recovery [8]. However, recovery of erectile function is not common, even in patients subjected to prostatectomy in which cavernous nerves were successfully preserved and in patients without toxic habits and disorders closely related to ED. Recently, it has been reported that this technique may cause erectile dysfunction in around 70% of cases [9]. These findings suggest the existence of additional factors which may modulate erectile function after prostatectomy. It would be interesting to analyze additional predictors with the ability to identify patients at long-term risk of ED after prostatectomy.

One of the main causes associated with ED is the reduction of the nitric oxide (NO)-dependent relaxing system [10]. During sexual stimulation, vascular and sinusoidal forces on the endothelium lead to sustained activation of the constitutive endothelial nitric oxide synthase (NOS3) enzyme in the endothelium. NO activates soluble guanylate cyclase (sGC), stimulating the formation of cyclic guanosine monophosphate(cGMP) [11]. cGMP is catabolized and removed by the phosphodiesterase 5 (PDE5). The NO-cGMP-PDE5 axis is then crucial for erection prior to intercourse as well as for the maintenance of pulmonary and cardiovascular health [12,13].

Different works have reported decreased expression of the NO/cGMP pathway and increased PDE5 expression in prostate cancer cell lines [14]. Restoration of the NO/cGMP pathway by PDE5 inhibitor treatment not only improved the antitumor effects of chemotherapeutic agents but was also shown to be useful in treating disorders after prostatectomy [15]. This restoration of the NO/cGMP pathway was observed in cancerous cells located in the prostate and in healthy cells from other tissues [16]. The NO-sGC-PDE5 axis is expressed differently in other cells than in endothelial cells. It has been recognized that changes in the NO-sGC-PDE5 system may, at the same time, be occurring in the penis and in other cells, tissues, and organs, suggesting that ED is an early signal of the risk of hypertension and cardiovascular diseases [17]. Different works have reported changes in the NO-sGC-PDE5 axis associated with ED beyond the penis, such as in plasma samples, endothelial progenitor cells, and leukocytes [18,19]. These findings suggest that changes in protein expression of the NO-sGC-PDE5 axis involved in ED may be reflected in other tissues beyond the penis, including buccal mucosa.

The buccal mucosa is comprised of the mucosal surfaces of the cheeks and lips, which form the anterolateral boundaries of the oral vestibule. The oral cavity has the advantage of being readily accessible for sampling with noninvasive techniques using buccal swabs [20]. Both constitutive NOS and PDE activity have been identified in the buccal mucosa, raising the possibility that in the buccal mucosa, the content of the NO-sGC-PDE axis may also reflect erectile functionality [21,22]. In the present work, we have determined the buccal mucosa content of proteins associated with the NO-cGMP-PDE5 axis, comparing them among patients with functional and dysfunctional erection one year after radical prostatectomy.

## 2. Materials and Methods

### 2.1. Patients and Buccal Mucosa Samples Recovery

Thirty-eight non-diabetic prostate cancer patients with normal functional erection who underwent programmed radical prostatectomy were included in the study. All patients underwent an interfascial nerve-sparing prostatectomy. The surgery was performed in an antegrade manner using clips and thermal energy. A validated Spanish version of the International Index of Erectile Function-Erectile Function Domain (IIEF-EFD) questionnaire was used to evaluate erectile function [23]. The IIEF-EFD score was successfully validated as a diagnostic tool that distinguishes between men without and men with ED and different ED severity classifications (i.e., no ED = EFD score 26–30; mild-moderate ED = EFD score 25–11; severe ED = score 6–10 [24]. After surgery, all patients had ED, and they were re-evaluated 6 months after the surgical intervention. Some patients who, at 6 months after surgery, had ED (IIEF-EFD score ≤ 25) were then treated with either PDE5 inhibitors, prostaglandin E1 therapy, or both. Buccal mucosa samples were collected 12 months after radical prostatectomy, where IIEF-EFD was evaluated again. Additional cofactors closely related to erectile function recovery, such as mental disorders, were considered. No patients showed anxiety or depression at 6 and 12 months after radical prostatectomy. Sample size calculation was based on an α risk = 0.05 and β risk = 0.20 and considering the experience in previous studies of protein expression of the NO-cGMP-PDE5 axis [18,19]. It was assumed that proteins between groups would reach a 15% change among them.

Samples were obtained from buccal mucosa using a light scraping with enzymes-treated cotton buccal swab (4N6FLOQSwabs™. Thermo Fisher, South San Francisco, CA) and placed on chemically treated FTA-cards (4473978. Thermo Fisher, South San Francisco, CA, USA). Proteins and genomic DNA were extracted from the FTA-card using a commercial kit according to the manufacturer’s instructions (Prepfiler forensic DNA extraction kit, 4463351. Thermo Fisher, South San Francisco, CA, USA).

All the included subjects gave fully informed consent, and the study was approved by the local ethics committee of clinical research of the Jiménez Díaz Foundation (EO 89/2014_FJD). The study was blind for the basic researchers, who determined the molecular parameters and performed the statistical analysis.

### 2.2. Interleukin (IL)-6 and IL-10 Determination

IL-6 and IL-10 levels were determined in buccal mucosa samples using commercial enzyme-linked immunosorbent assay (ELISA) kits. As previously reported, ELISA kits for IL6 (Quantikine ELISA Human IL-6. D6050; R and D Systems, Abingdon, Oxfordshire, UK) and IL10 (Quantikine ELISA Human IL-10. D1000B; R and D Systems) were performed following the manufacturer’s instructions, using an assay sensitivity of 0.7 pg/mL and 3.9 pg/mL, respectively. The intra-and inter-assay variation coefficients were 1.7–4.4% and 2.0–3.7% for IL-6 and 2.5–6.6% and 5.6–7.6% for IL-10.

### 2.3. Determination of Buccal Mucosal Levels of NOS3, β1-Subunit of sGC and PDE5

In buccal mucosa samples, protein levels of NOS3, β1-subunit of sGC, and PDE5 were analyzed by dot-blot. Due to the relative low level of total proteins recovered by scraping buccal mucosa, it could not be possible to use Western blot technique. Protein samples obtained from the sample cards were solubilized in Laemmli buffer containing 2-mercaptoethanol. Equal amounts of proteins (10 μg/sample) estimated by bicinchoninic acid reagent (Pierce, Rockford, IL, USA) were loaded for each sample. Dot-blots analysis was developed with a monoclonal antibody against NOS3 (#N30020; 1:1500 Transduction Laboratories, San Diego, CA, USA), β1-sGC (#210–786; 1:1500; Alexis laboratories, San Diego, CA, USA) or PDE5 (#524583; 1:1500 Calbiochem, La Jolla, CA, USA). The constitutive protein β-actin was also determined by using a specific monoclonal antibody (#A5441; 1:2500; Sigma Chemical Co., St Louis, MO, USA). Blots were incubated with the first antibody against NOS3, β1-sGC, PDE5 or β-actin for 1 h at room temperature and, after washing, with secondary antibody (horseradish peroxidase-conjugated IgG antibody) at 1:2500 dilution for another hour. The protein signal was developed by chemiluminescence (ECL; GE Healthcare, Little Chalfont, Buckinghamshire, UK) and detected using the iBright Imaging System (iBright FL100, Thermofisher Scientific, Waltham, MA USA).

### 2.4. Analysis of the Rs3806808 PDE5A Gene Polymorphism

The rs3806808 polymorphism of the PDE5A gene was genotyped by TaqMan assay. TaqMan primers were obtained through the TaqMan™ Gene Expression application (Thermofisher.com). Probes were labeled with VIC or FAM dye. Real Time-PCR amplifications were performed in an ABI PRISM 7900 HT Sequence Detection System (Applied Biosystems). Genotypes were differentiated by analyzing the fluorescence levels of PCR products using an ABI PRISM 7900HT Sequence Detector (Applied Biosystems). Genotyping was performed blindly in the central services of Madrid Science Park. 

### 2.5. Statistical Analysis

Variables were expressed as mean ± error standard mean. Data did not show normal distribution after performing the Kolmogorov–Smirnov test. Statistical analysis between patients with functional and dysfunctional erection was performed using the non-parametric Mann–Whitney test. Distribution of genotypes and alleles of the PDE5 gene polymorphism were evaluated using the Chi-square test. A *p*-value < 0.05 was considered statistically significant. Statistical analysis was performed using the SPSS program (version 25.0).

## 3. Results

Based on the IIEF-EFD score, included patients were divided into two groups: patients with functional (IIEF-EFD score ≥ 26) and patients with ED (IIEF-EFD score < 26) (Table 1). It is noted that in patients who were pharmacologically treated against ED, the IIEF-EFD questionnaire was evaluated considering such treatment. 

As Table 1 shows, IIEF-EFD was significantly lower in patients with ED. Age was similar among both groups of patients (Table 1). The frequency of preservation of neurovascular bundles and the prostatectomy procedures were not statistically different among patients showing dysfunctional and functional erection (Table 1). Total testosterone and free testosterone plasma levels were also similar among patients with functional and dysfunctional erection (Table 1). 

One year after radical surgical prostatectomy, ED patients tended to be slightly less frequently treated with PDE5 inhibitors than patients with erectile functionality, although it did not reach statistical significance (Table 1). Patients with functional and dysfunctional erections were treated with similar frequency with prostaglandin E1 or with dual treatment with PDE5 inhibitors and prostaglandin E1 (Table 1). More frequently, ED patients were without these treatments than patients that have recovered functional erection one year after surgery (Table 1).

In buccal mucosa samples, the inflammatory state was also evaluated by measuring the content of pro-inflammatory cytokine IL-6 and the anti-inflammatory cytokine IL-10. Although buccal mucosa levels of IL-6 tended to be lower in the patients with normal erectile functionality than in those with ED, it did not reach statistical differences (Table 2). Neither the buccal mucosa levels of IL-10 nor the IL-10/IL-6 ratio were statistically different between patients with and without ED (Table 2).

### 3.1. Changes in Buccal Mucosa of the Nitric Oxide System and Its Relationship with the IIEF-EFD Score 

**Figure 1:**
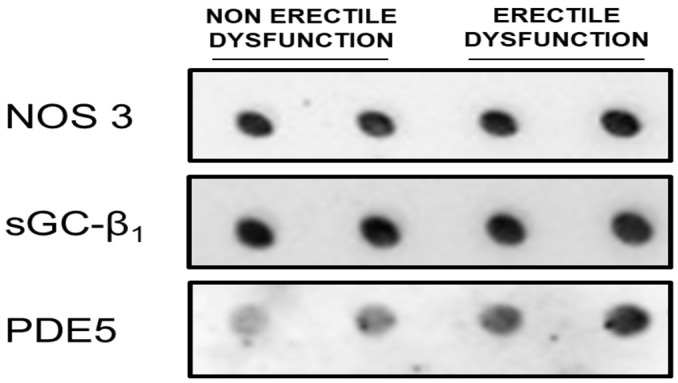
Representative dot-blots showing the expression of nitric oxide synthae3 (NOS3), the β1 subunit of soluble guanylate cyclase (sGC-β1), and phosphodiesterase 5 (PDE5) in buccal mucosa samples obtained from patients with functional and dysfunctional erection one year after radical prostatectomy.

**Figure 2:**
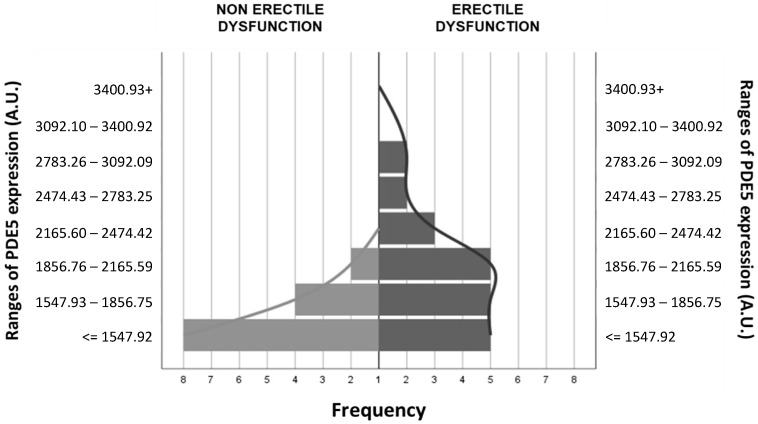
Histogram showing the frequency of phosphodiesterase 5 expression ranges in buccal mucosa samples obtained from patients with functional and dysfunctional erection one year after radical prostatectomy. Abbreviations. PDE5: phosphodiesterase 5; A.U: densitometric Arbitrary Units.

Protein expressions of NOS3 and β1-sGC were similar in the buccal mucosa of patients with functional and dysfunctional erection (Table 2 and Figure 1). However, PDE5 levels were significantly higher in patients with ED compared to patients with normal erectile function (Table 2 and Figure 1). The distribution of PDE5 expression ranges in buccal mucosa samples obtained from patients with functional and dysfunctional erections one year after radical prostatectomy is shown in Figure 2.

ED severity may be graded as mild, moderate, or severe according to the reached IIEF-EFD. In fact, an IIEF-EFD score ≥ 26 indicates no ED, a score questionnaire of 11–25 indicates mild-moderate ED, and 6–10 indicates severe ED.

One year after the radical prostatectomy of the 23 patients with ED, 43.48% of them had severe ED, and 56.52% of them had mild-moderate ED (Table 3). Among these two groups of ED patients, results did not show statistical differences in the buccal mucosa content of IL-6, IL-10, and in NOS3, β1-sGC, and PDE5 protein expression (Table 3). However, in buccal mucosa, the IL-10/IL-6 ratio was significantly higher in mild-moderate ED patients than in those with severe ED.

### 3.2. Rs3806808 PDE5A Gene Polymorphism

An analysis was undertaken to see whether changes in buccal mucosa PDE5 expression between patients with and without ED could be related to genetic variability. The genotype and allelic frequency of rs3806808 polymorphism in the PDE5A gene were analyzed. The allelic frequency of A and C was similar between patients with and without ED one year after radical surgical prostatectomy (Table 4). Genotyping distribution of the possible variants (AA, AC, CC) in the rs3806808 PDE5A polymorphism was not different between patients with and without ED (Table 4).

Moreover, a possible association between positive pharmacological responses to PDE5 inhibition and the rs3806808 PDE5A polymorphism was analyzed. ED patients subjected to PDE5 inhibitor treatment had a higher content of PDE5 protein than patients under PDE5 inhibitor treatment who recovered erectile functionality one year after radical surgical prostatectomy (Table 5). However, allelic frequency and genotype distribution of the rs3806808 PDE5A polymorphism were similar among patients subjected to PDE5 inhibitor treatment, regardless of whether or not they recovered erectile function (Table 5).

## 4. Discussion

The present work shows, in our knowledge for the first time, that one year after radical prostatectomy, buccal mucosa content of PDE5 protein was significantly lower in the patients showing ED compared to those from patients who had recovered erectile functionality. 

PDE5 activity is involved in ED by increasing cGMP metabolism. PDE5 protein is expressed in the corpus cavernous and also in other organs and cells such as the digestive apparatus, heart, lung, smooth muscle, mononuclear cells, and prostate, among other localizations [19,25]. The results supported the presence of PDE5 in buccal mucosa; its expression was increased in patients with ED one year after the radical prostatectomy. This suggests that, in terms of PDE5 expression, ED could be reflected in other tissues, such as buccal mucosa, supporting the finding that ED does not only take the form of functional disability of penis erection. Several works have reported a high prevalence of ED in the patient population suffering from cardiovascular diseases [26]. Moreover, it was reported that patients with cavernous arterial insufficiency have a significantly greater risk of developing coronary artery disease [27]. Thompson et al. reported a significant positive association between incidents of ED and subsequent angina, myocardial infarction, and stroke [28].

The fact that one year after radical prostatectomy, the expression levels in the buccal mucosa of other enzymes involved in the NO-related system, i.e., NOS3 and the β1-sGC, were similar between patients who recovered one year after radical prostatectomy and those who did experience erectile functionality may support the specificity of the results. 

According to IEEF-EFD questionnaire, ED severity was not associated with differences in the buccal mucosa content of PDE5. This suggests that changes in PDE5 content in the buccal mucosa could be possible marker of normal erectile functionality without discriminating ED degree.

With the present experimental design, it is not possible to know the molecular mechanism involved in the higher PDE5 protein expression in the buccal mucosa of ED patients. In patients with diabetic cardiomyopathy, an association was reported between PDE5 inhibitor treatment and reduced inflammation. PDE5 inhibition also reduced levels of inflammatory-associated proteins in plasma from ED patients [19]. Therefore, changes in the local inflammatory state could influence the content of PDE5 in the buccal mucosa. However, the results demonstrated that buccal mucosa content of IL-6 and IL-10 were not different among the patients with and without ED one year after radical prostatectomy. Moreover, although in buccal mucosa, the IL-10/IL-6 ratio was higher in mild-moderate ED patients than in those showing severe ED, PDE5 levels were similar among them. These results diminished the involvement of the buccal mucosa inflammatory state in the regulation of PDE5 expression.

There are also some controversial results; some authors have observed an inverse association between testosterone levels and ED [29,30]. Testosterone levels have been associated with changes in PDE5 expression by itself, and testosterone has also been detected in salivary samples [31]. Unfortunately, in the present study, salivary testosterone levels were not measured. However, the fact that both total and free plasma testosterone levels were similar among patients with and without ED may diminish the involvement of testosterone in the observed differences in PDE5 expression. Other mechanisms and mediators associated with the regulation of PDE5 expression, such as nitrosylation PDE5 degree, should be analyzed. 

Several studies have supported the potential positive effect of PDE5 inhibitors on erectile function recovery after radical prostatectomy [32]. Despite the evidence that PDE5 inhibitor treatment may be successful in men with monotherapy failure, several findings suggest the need to perform double-blind, randomized controlled trials to ensure the benefits, optimal dosage, possible adverse effects, and acceptability to patients of these treatments [33]. The fact of identifying specific changes in the expression levels of PDE5 in patients with persistent ED one year after radical prostatectomy may suggest two important aspects: 1. the potential existence of endogen factors that prolong the alteration of PDE5 expression over time, and 2. it would be plausible to consider that endogen factors would have the ability to diminish the efficacy of PDE5 inhibitor treatment. Interestingly, it has been described that PDE5 expression and response to PDE5 inhibition treatment may be specifically determined by different genetic polymorphisms [34,35]. In this regard, the presence of the rs3806808 PDE5A gene polymorphism affected the binding affinity of PDE inhibitors, reducing the response to NO [13]. In the present work, genotype and allelic frequencies in the rs3806808 polymorphism of the PDE5A gene were not different between patients with functional and dysfunctional erection one year after prostatectomy. Moreover, there were patients under PDE5 inhibitor treatment who continued to have ED. It has been reported that PDE5 inhibitor therapy failed to restore erection for sexual intercourse in almost 32% of ED patients with selective PDE5 inhibitors [36]. Patients under treatment with PDE5 inhibitors continued experiencing ED one year after radical prostatectomy, and they had increased buccal mucosa PDE5 levels as compared with those patients also treated with PDE5 inhibitors who recovered erectile functionality. However, patients under treatment with PDE5 inhibitors have similar genotypes and allelic frequency in the rs3806808 PDE5A gene polymorphism independently of their erectile functionality. Therefore, rs3806808 polymorphism may not be associated with variation expression of PDE5 protein in buccal mucosa or with pharmacological response to PDE5 inhibitors one year after radical prostatectomy. However, it does not discard the possibility that other polymorphisms not analyzed in the present study are involved in differential PDE5 expression and decreased response to PDE5 inhibitor treatment.

The main strength of this work was to open the possibility that specific changes in the expression levels of proteins involved in the ED pathophysiology could be identified in tissue with non-invasive accessibility, such as buccal mucosa, in patients with persistent ED one year after radical prostatectomy. This finding may be used to identify quickly and non-invasively those patients with persistent ED after radical prostatectomy who would benefit from genetic screening to identify potential genetic polymorphisms that underlay alterations of PDE5 expression and that may be influencing the response to PDE5 inhibitor treatment. It opens the possibility of, in the future, arriving at personalized solutions to establish more rational use of treatment. However, it is also important to point out several limitations to the study. A main question raised from the present results is whether changes in buccal mucosa of PDE5 expression could be used as a predictive marker for detection in patients with ED after radical prostatectomy. Unfortunately, buccal mucosa samples were only collected one year after radical prostatectomy. Therefore, it was not possible to analyze the PDE5 expression in the buccal mucosa at different times after radical prostatectomy. In addition, buccal mucosa could express different PDE5 levels than the penis. Correlation between PDE5 levels in buccal mucosa and penis should have been performed to clarify the potential relationship in PDE5 expression among both tissues. However, ethical aspects related to patients’ erectile tissue biopsies did not allow us to analyze them. On the other hand, only some patients received medical treatment for the symptomatic management of erectile dysfunction, which may modify the expression of proteins associated with the NO-cGMP-PDE5 axis. This should be considered as a potential confounding factor. Furthermore, in the present study, only rs3806808 PDE5A gene polymorphism was analyzed. However, it would be plausible to think that different factors, such as pharmacological concomitant treatments or clustering with other polymorphisms, may modify the impact of the above-mentioned SNPs on protein expression of PDE5 and, therefore, respond to PDE5 inhibitor treatment.

## 5. Conclusions

One year after radical prostatectomy, patients with persistent ED showed increased PDE5 levels in buccal mucosa compared to those patients who recovered erectile functionality. The allelic and genotype frequency of the rs3806808 PDE5A gene polymorphism was associated with neither PDE5 expression changes in the buccal mucosa nor penis erection improvement by PDE5 inhibition in patients with radical prostatectomy. Follow-up studies are required to clarify if increased PDE5 expression in buccal mucosa could be used as a predictive marker for ED after radical prostatectomy.

## Figures and Tables

**Table 1:** Clinical and surgical characteristics of the patients who underwent radical prostatectomy.

Variables		Functional Erection (n = 15)	ED (n = 23)	*p* Value
**IIEF-EFD score**		26.93 ± 0.21	12.35 ± 1.44	<0.001
**Age** (y)		61.27 ± 1.44	60.30 ± 1.12	0.631
**Neurovascular bundle preserved N** (%)	None	0 (0.0)	4 (17.4)	0.264
	Left	4 (26.7)	7 (30.4)	
	Right	5 (33.3)	6 (26.1)	
	Both	6 (40.0)	6 (26.1)	
**Radical prostectomy procedure**	Open	10 (66.7)	12 (52.2)	0.376
	Laparoscopy	4 (26.7)	10 (43.5)	0.294
	Robotic	1 (6.7)	1 (4.3)	0.754
**Total testosterone** (ng/mL)		4.5 ± 0.8	4.9 ± 0.6	0.787
**Free testosterone** (pg/mL)		44.8 ± 2.1	46.6 ± 0.6	0.760
**Pharmacological treatment N** (%)	None	1 (6.7)	8 (34.8)	0.046
	PDE5 inhibitors	8 (53.3)	6 (26.1)	0.089
	PGE_1_	4 (26.7)	6 (26.1)	0.968
	Both	2 (13.3)	3 (13.0)	0.979

Results of quantitative variables are represented as mean ± standard error of the mean (SEM). Categorical variables are represented as frequency (percentage). The non-parametric Mann–Whitney test was used to compare quantitative variables. The chi-square test was used to compare categorical variables. Abbreviations.—IIEF-EFD: International Index of Erectile Function-Erectile Function Domain; y: years; PDE5: Phosphodiesterase 5; PGE1: Prostaglandin E1.

**Table 2:** Buccal mucosa content of IL-6, IL-10 and enzymes related to the NO system.

Variables	Functional Erection (n = 15)	ED (n = 23)	*p* Value
**IL-10** (pg/μg tissue protein)	1.45 ± 0.15	1.10 ± 0.12	0.225
**IL-6** (pg/μg tissue protein)	0.12 ± 0.02	0.24 ± 0.09	0.662
**IL-10/IL-6 Ratio**	14.03 ± 2.12	13.79 ± 4.36	0.207
**NOS3** (A.U)	1606.78 ± 87.28	1509.70 ± 111.51	0.953
**SGC-β1** (A.U)	1936.54 ± 99.67	1723.69 ± 111.75	0.344
**PDE5** (A.U)	1569.23 ± 79.37	2045.30 ± 125.71	0.004

Results are represented as mean ± standard error of the mean (SEM). The non-parametric Mann–Whitney test was used to compare variables Abbreviations. IL (Interleukin); NOS3: Nitric Oxide Synthase 3; sGC-β1: soluble guanylate cyclase β1 subunit; PDE5: Phosphodiesterase 5; A.U: Densitometric Arbitrary Units.

**Table 3:** Buccal mucosa content of IL-6, IL-10, and enzymes related to the NO system.

Variables	Mild-Moderate ED (n = 13)	Severe ED (n = 10)	*p* Value
**IIEF-EFD score**	17.23 ± 1.44	6.0 ± 0.39	<0.001
**IL-6** (pg/μg tissue protein)	0.25 ± 0.13	0.20 ± 0.04	0.141
**IL-10** (pg/μg tissue protein)	1.18 ± 0.17	0.90 ± 0.06	0.112
**IL-10/IL-6 ratio**	16.87 ± 5.83	6.08 ± 2.36	0.031
**NOS3** (A.U)	1645.30 ± 125.14	1333.50 ± 191.45	0.321
**sGC-β_1_** (A.U)	1821.67 ± 119.55	1596.32 ± 205.53	0.385
**PDE5** (A.U)	2083.99 ± 187.01	1995.01 ± 166.54	0.804

Results are represented as mean ± standard error of the mean (SEM). The non-parametric Mann–Whitney test was used to compare quantitative variables. Abbreviations. IIEF-EFD: International Index of Erectile Function-Erectile Function; IL (Interleukin); NOS3: Nitric Oxide Synthase 3; SGC-β1: β1 subunit of soluble guanylate cyclase; PDE5: Phosphodiesterase 5; A.U: Densitometric Arbitrary Units.

**Table 4:** Comparison of genetic variant frequencies in the rs3806808 PDE5A gene polymorphism among erectile functional and ED patients.

Gene	Variants	Functional Erection (N = 15)% (N)	ED (N = 22)% (N)	*p* Value
**PDE5A**	AA	40.0 (6)	45.5 (10)	0.212
	AC	46.7 (7)	54.5 (12)	
	CC	13.3 (2)	0 (0)	
	Allele A	63.3 (19)	72.7 (32)	0.391
	Allele C	36.7 (11)	27.3 (12)	

Distribution of genotypes and alleles of the PDE5 gene polymorphism were evaluated using the Chi-square test. Abbreviations: PDE5: Phosphodiesterase 5.

**Table 5:** Comparison of PDE5 content in the buccal mucosa and genetic variant frequencies in the rs3806808 PDE5A gene polymorphism among radical prostatectomy patients with functional and dysfunctional erection treated with PDE5 inhibitors.

Variables		Functional Erection (N = 10)	ED (N = 9)	*p* Value
**PDE5 content (A.U)**		1578.3 ± 103.59	2021.91 ± 163.55	0.041
**Gene**	**Variants**	**Non dysfunction (N** **=** **10)% (N)**	**Dysfunction (N** **=** **9)% (N)**	***p* Value**
**PDE5A**	**AA**	40.0 (4)	57.1 (4)	0.606
	**AC**	50.0 (5)	42.9 (3)	
	**CC**	10.0 (1)	0 (0)	
	**Allele A**	65.0 (13)	78.6 (11)	0.393
	**Allele C**	35.0 (7)	21.4 (3)	

PDE5 content results are represented as mean ± standard error of the mean (SEM). The non-parametric Mann–Whitney test was used to compare PDE5 content. Genotypic and allelic distributions of the rs3806808 PDE5 gene polymorphism are presented as percentage and frequency. The distribution of genotypes and alleles of the PDE5 gene polymorphism was evaluated using the Chi-square test. Abbreviations. PDE5: Phosphodiesterase 5; A.U: Densitometry Arbitrary units.

## Data Availability

The data that support the findings of this study are available on reasonable request from the corresponding author.
